# Entering the Classroom: Do Newcomers Experience More Peer Victimization than Their Established Peers?

**DOI:** 10.1007/s10802-024-01225-6

**Published:** 2024-07-13

**Authors:** Essi-Lotta Tenhunen, Sarah Malamut, Patricia McMullin, Tiina Turunen, Takuya Yanagida, Christina Salmivalli

**Affiliations:** 1 INVEST Research Flagship, Department of Psychology, University of Turku, Turku, 20500 Finland; 2 INVEST Research Flagship, Department of Sociology, University of Turku, Turku, Finland; 3 Department of Developmental and Educational Psychology, Faculty of Psychology, University of Vienna, Vienna, Austria

**Keywords:** Ingroup bias, Newcomer, Peer victimization, School change, Student mobility

## Abstract

Students changing classrooms or schools may face challenges from entering a new peer context without friends and standing out from the crowd as *newcomers*. Two studies examined whether newcomer status predicts peer victimization at school, exploring several potential moderating factors (e.g., social anxiety, immigrant background and having good friends in the classroom) (Study 1: *n* = 6,199; *M*_age_=12.53) and whether being victimized as a newcomer varied based on the different reasons for mobility (e.g., parental dissolution, residential move, previous victimization, changing into a more suitable school) (Study 2: *n* = 58,738). In both studies, newcomers reported higher peer victimization compared to established students. Having good friends in the classroom was found as a protective factor in Study 1, being the only statistically significant moderator. All reasons for mobility, except changing into a more suitable school, predicted slightly higher peer victimization in Study 2, with the highest risk for those changing schools due to previous peer victimization experiences.

Student mobility, defined as nonpromotional school or classroom change, is a widespread phenomenon experienced by many students each year. As students establish ties with their peers at school, and such relationships are increasingly valued as they grow older (Larson & Richards, 1991), losing social ties and standing out as a newcomer in the new peer group may give rise to challenges in peer relations. In particular, newcomers may be vulnerable to bullying victimization (being repetitively and intentionally harmed or hurt by a more powerful peer), which is suggested to be a group phenomenon by nature (Salmivalli, 2010). The aim of this multi-sample study was to investigate whether newcomers report more frequent peer victimization compared to established students, and whether this depends on demographic (e.g., immigrant background), individual (social anxiety) and interpersonal (having good friends in classroom) factors (Study 1) or on the initial reason for mobility (Study 2).

An important component for group harmony is interpersonal and intragroup compatibility. As suggested by the influence-compatibility model (Laursen & Veenstra, 2021), peer influence occurs within classrooms or peer groups to increase similarity among individuals, which in turn increases within-group compatibility. Newcomers who enter an established classroom have not been exposed to the peer group’s pre-existing social norms and dynamics and therefore have not had a chance to become compatible group members. Before newcomers have integrated into the peer group, they may therefore be viewed as incompatible group members, or “misfits”. According to social misfit theory (Wright et al., 1986), individuals deviating from what is considered normative in the group are at heightened risk for rejection by group members. Indeed, previous studies have found that early adolescents who violated norms of the peer groups (e.g., classroom norms of aggression) were more likely to be victimized (Bass et al., 2022). This underscores the importance of social norms in shaping peer interactions and the consequences of deviating from the peer group in adolescence. Newcomers may therefore be at risk for peer problems, including victimization, until they have integrated in the classroom. Yet another theoretical perspective that may inform newcomers’ adjustment is social identity theory (Tajfel & Turner, 1986) which suggests that individuals tend to favor ingroup members and even mistreat those belonging to outgroups. The classroom can be considered a social group with which the established students identify, and group membership might become especially salient when a newcomer – at that point an outgroup member – enters the group. The outgroup effect is likely to be pronounced when the newcomer represents another social category also in other respects, for instance having an immigrant background.

Previous studies on peer victimization during school transitions mostly focused on normative transitions characterized by predictable promotional changes in educational phases (e.g., moving from elementary to middle school together with one’s age cohort). These studies often found decreased peer victimization (e.g., Farmer et al., 2011; Pellegrini & Long, 2002; Wang et al., 2016) or no change in victimization (Lorijn et al., 2024) after the transition. Non-normative school transitions, encompassing unexpected disruptions in a student’s academic journey and standing out as the only new student in the classroom or school, have received less attention from the peer victimization perspective. Instead, studies have examined other negative consequences, such as increased risk for witnessing serious school violence or weapon carrying in school (Foster & Brooks-Gunn, 2012), decreased school performance (Li et al., 2019; Pribesh & Downey, 1999), not completing upper secondary education (McMullin et al., 2021), loss of social connections (Pribesh & Downey, 1999; South & Haynie, 2004), mental health problems (Li et al., 2019), and externalizing behavior (e.g., Adam & Chase-Lansdale, 2002; Coley et al., 2013; South & Haynie, 2004; see also review by Cotton, 2016).

At least four studies examined the association between newcomer status (caused by a non-normative classroom or school transition) and victimization. Due to differences in their designs and methods, it is difficult to draw conclusions based on these studies, or to even compare them. One study (Vandell et al., 2021) found student mobility to be associated with subsequent loneliness, whereas multiple moves increased the likelihood of peer victimization in grade five. However, these effects were not robust enough to remain significant after correcting for multiple comparisons. According to another study (Carson et al., 2013), peer victimization was predictive of mobility, but mobility did not predict victimization. Changing schools within the school district (as long-distance movers were excluded) actually led to a decreasing average level of victimization (though this occurred across the entire sample, not solely among those previously victimized). Both findings were observed among the middle schoolers, but not in the high school sample. A third study (Salmivalli et al., 1998) found that victimization was equally stable (from the sixth to the eighth grade) among middle school students who had either made the school transition (when starting grade 7) together with their former classmates and among those who had entered classrooms with new classmates. However, the study examined rank-order stability, not victimization trajectories over time. Finally, Rambaran and colleagues (2019) found that being a newcomer was generally not associated with victimization, but the direction of the association in “unstable” schools (e.g., schools with frequent changes in classroom compositions) indicated newcomer’s higher risk to report peer victimization.

The above studies overlooked both the reasons for mobility (only Carson et al. focused on previous victimization as the reason for mobility, but the follow-up confounded students who had moved for this reason with those who changed schools for other reasons) and potential moderators (except Vandell et al., who tested family income and mother’s supportive parenting). To tackle the gaps in the previous literature, the present study uses a Finnish basic education school sample (where the student mobility rate is around 5%), tests several moderators, controls for multiple individual and family factors, as well as examines if some of the initial reasons for mobility predict peer victimization more strongly than others.

## Potential Moderators

To shed light on individual characteristics or circumstances that may alleviate or exacerbate newcomers’ risk for peer victimization, there are several relevant moderators. Previous studies on student mobility have found that the linkage between student mobility and a less central position in friendship networks (South & Haynie, 2004) as well as decreased academic achievement and depressive symptoms (Li et al., 2019) are moderated by age at the time of mobility. It has been suggested that student mobility matters more for older youth than for younger children (Li et al., 2019; South & Haynie, 2004) due to developmental changes in peer relations. Overall, peer relations are more important for adolescents than younger children, for whom family relationships are still more central (Larson & Richards, 1991). Also, group conformity is relatively high, whereas group permeability (willingness to accept new members to one’s ingroup) is relatively low in early and middle adolescence (Gavin & Furman, 1989). Newcomer victimization may therefore be more prevalent among middle schoolers, as compared with elementary school students.

Furthermore, it is crucial to account for living with one parent (Pribesh & Downey, 1999) and having an immigrant background (Kuyvenhoven & Das, 2022; South & Haynie, 2004) as they are associated with an increased likelihood of changing residence and, therefore also the classroom and school. Single parenting has also been linked to scarcer parental resources, including less time available for parental monitoring, which is associated with peer victimization (Hartinger-Saunders et al., 2012). Moreover, children with immigrant backgrounds are overall at a higher risk of being victimized by their peers (Strohmeier et al., 2011). Moreover, families with immigrant backgrounds and single-headed households tend to have lower socioeconomic positions and live in disadvantaged areas where behavioral and social-emotional problems are more common among children (McLoyd, 1998).

Additional characteristics that could moderate the association are social anxiety and having good friends in the classroom. Social anxiety (Forbes et al., 2018) and friendlessness (Salmivalli & Isaacs, 2005) are both linked to peer victimization. Student mobility is often associated with various stressors, such as immigration (Kuyvenhoven & Das, 2022), family-related challenges, or family dissolution (Pribesh & Downey, 1999), former experiences of peer victimization (Carson et al., 2013), and loss of social ties (South & Haynie, 2004). Individuals may exhibit symptoms of anxiety while coping with such stressors (Young & Dietrich, 2015). Since individuals with social anxiety tend to feel uncomfortable in social situations and meeting new people (Van Zalk et al., 2011), newcomers with such symptoms are likely to find the integration process into the new classroom more challenging, while peers might see them as “easy” targets.

The essential role of friendships has been underlined in previous research on student mobility (e.g., Pribesh & Downey, 1999; South & Haynie, 2004), suggesting that newcomers may face negative consequences due to a lack of friendships in the new environment. Indeed, having friendships has been found to protect children with other risk factors (e.g., internalizing symptoms) from victimization (Hodges et al., 1999). Thus, it is important to examine whether having friends protects newcomers from peer victimization in new classrooms.

## Reasons for Student Mobility

Non-normative student mobility can result from within and between-country migration or solely changing classrooms or schools while staying in the same residence. The causes of residential mobility are diverse, meaning that they can vary from negative (i.e., eviction, family dissolution, parental death, a child taken to custody) to positive (i.e., moving to a larger home or better neighborhood, moving for parent’s new career opportunities) life events (Tønnessen et al., 2016). Likewise, student mobility without residential change may be a consequence of negative as well as positive reasons, such as changing schools because of peer victimization or changing to a more suitable school providing more tailored opportunities (e.g., weighted curriculum) or extra support (e.g., special education) that fit the child’s needs. Weighted curriculum refers to an educational approach where certain subjects or areas of study are given greater emphasis or importance compared to others within the overall curriculum.

Family migration stands out as a common reason for non-normative student mobility, often associated with adverse consequences (Pribesh & Downey, 1999; Foster & Brooks-Gunn, 2012). While some negative outcomes might be linked to family circumstances prompting the move, research suggests that mobility itself can have unique effects. For instance, Pribesh and Downey (1999) noted a significant negative impact on school performance across various household types, indicating mobility’s independent influence.

Peer interaction in the previous school can also drive student mobility, especially when students face challenges such as peer victimization or bullying. Studies investigating the persistence of victimization after a change in classroom settings offer slightly mixed findings. While some suggest victimization persists despite relocation (Salmivalli et al., 1998), others indicate a reduction in victimization for students transitioning to a new school (Carson et al., 2013).

Beyond issues related to peer relations, reasons for student mobility may include parental career changes, financial considerations, or family composition alterations. Norford and Medway (2002) explored how such factors affected students’ emotional well-being and engagement in extracurricular activities, finding some indication that students who moved due to parental divorce showed lower involvement in extracurriculars in the new school. Further research is needed to understand the inconsistencies arising from previous literature and to gain insight into how student mobility resulting from victimization vs. other reasons affects the situation in the new context.

## The Finnish School Context

Finnish basic education lasts nine years, consisting of six years in elementary school (grades 1 to 6) and three years in middle school (grades 7 to 9). The students typically begin the first grade the year they turn seven, in the school located in their neighborhood (in urban areas) or municipality (in rural areas). The classroom composition often remains the same through the elementary school grades but often changes in the transition from elementary to middle school (the transition from the 6th to the 7th grade). Elementary and middle schools are either completely different schools in distinct locations, or, when combined (elementary and middle grades in the same school), often in separate buildings. Most Finnish children enroll in education offered by the public sector (Ministry of Education and Culture, 2022). However, besides regular education, other options with tailored curriculum exist within the public sector (e.g., supported education for children with disabilities or weighted curriculum with more emphasis on subjects such as sports, music, mathematics, or languages).

To access the weighted education, students are evaluated based on their academic performance, skills, and sometimes interviews to determine their eligibility for admission into these specialized educational tracks. Thus, the students on the weighted education classrooms share a common interest on the main subject but also tend to have similar family background, whether culturally, financially, or socioeconomically. This homogeneity may decrease incompatibility challenges, as the selected students share similar perspectives and experiences but also because they often enter the classrooms simultaneously, rather than one student entering a pre-established group.

## The Present Studies

Despite theoretical reasons to expect that newcomer status is associated with an increased risk for peer victimization, there is a dearth of empirical evidence. The few studies investigating the topic have not considered demographic characteristics associated with changing schools or disentangled the role of the initial reasons for residential mobility on peer victimization. Thus, the key question remains: does the newcomer status as such, or some other, related factor make victimization experiences more likely among students who recently entered a new classroom or school? The present studies provide a nuanced view of the association between newcomer status and victimization. In Study 1, we investigate the role of student mobility on peer victimization while controlling for *gender, externalizing behavior (i.e., peer nominated bullying behavior), age, one-parent household*, *immigrant background*, *social anxiety, and having friends in the classroom.* In addition to controlling for these key variables, Study 1 also examines whether the latter five variables buffer or exacerbate the association between being a newcomer and victimization. In Study 2, we delve into the reasons for student mobility (i.e., *being victimized or having bullied others in the previous school, parental union dissolution, relocating with family, changing to a more suitable school, or some other reason*), examining if some of these reasons are more likely than others to be associated with peer victimization in the new context.

## Study 1. The Association Between Newcomer Status and Victimization

Study 1 tests two main hypotheses. First, newcomer status is expected to be associated with an increased risk of victimization. If the association between student mobility and peer victimization was explained by ingroup favoritism and perceiving the new student as an incompatible “misfit”, we would expect to see a unique link from mobility to victimization over and above the effects of anxiety and (not) having good friends in the classroom, regardless of the child’s gender, externalizing behavior, age, immigrant status, and whether or not the child is living with one parent (H1). Second, we further investigate whether this risk only exists in the school year when the newcomers first entered the new classroom, or whether it persists through the following school year. We expect to see newcomer victimization in the year when mobility happens but not in the next academic year (H2) as the students integrate into the new classroom, lose their newcomer status, or find a group to belong to. Finally, we explore whether the association between newcomer status and victimization depends on the presence of additional vulnerability factors by examining living with one parent, immigrant background, being socially anxious, and (not) having good friends in the classroom as moderators. We also test moderation by age, to examine whether newcomer status is a stronger predictor of victimization among students in middle school, as compared with those in elementary school.

## Methods

### Participants and Procedures

**Fig. 1:**
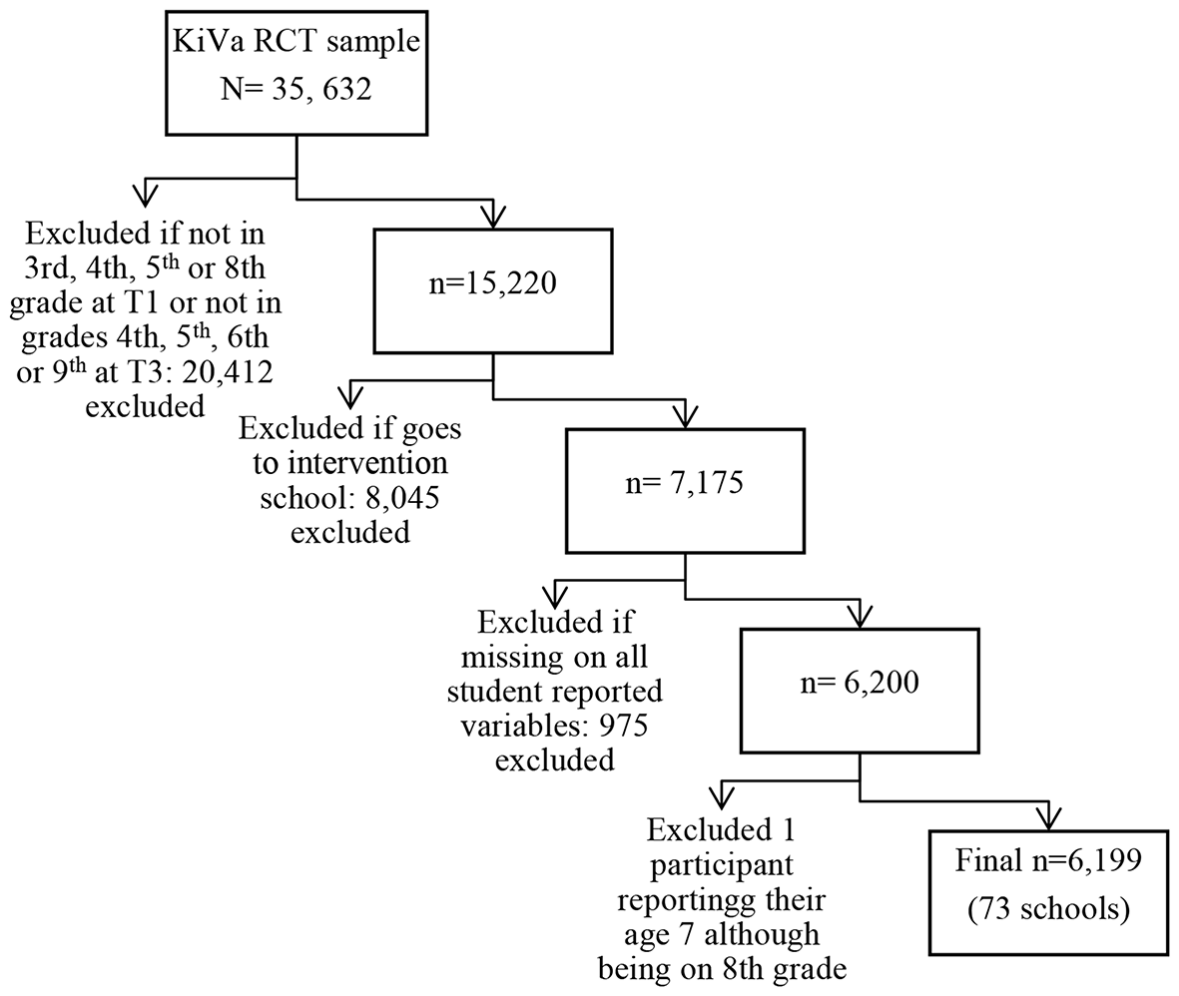
The steps to achieve the analytical sample (Study 1)

The study utilizes the first (T1) and third (T2 in the present study) waves of data (collected one year apart) from the KiVa randomized controlled trial (RCT) conducted in 2007-08 (elementary school, grades 3 to 5 at wave 1) and 2008-09 (middle school, grades 7 to 9 at wave 1). Parental consent was obtained through information letters including a consent form, and students with active parental consent were included in the study (see Kärnä et al., 2011 and Kärnä et al., 2012 for detailed description). The second wave was omitted as peer victimization questions were different among elementary vs. middle school students in that wave. The seventh graders at T1 (who were eighth graders at T2) were excluded because in the middle school transition, most students change schools simultaneously (i.e., normative transition) and the classrooms are often mixed. The sixth and ninth graders were excluded because they were about to leave the school soon after T1 data collection. Out of the 742 schools, 55% were intervention schools; those were excluded to minimize the potential impact of an anti-bullying intervention on our results. One student was excluded because they reported their age as 7, although they were in eighth grade. Figure 1 shows the steps to achieve the final analytical sample. The first wave of data (T1) was collected in May 2007 (in elementary schools) and May 2008 (in middle schools), and the third wave (T2 in the present study) one year later.

In total, 6,199 students participated in the study (*M*_*age*_=12.53; *SD* = 2.09, at T1). Descriptive statistics, along with information on missingness, are provided in Table 1. Out of all T1 participants, 49% were boys and 4.77% were newcomers. The measures of family background show that 8.33% had an immigrant background and 25.32% were living with one parent. Both an immigrant background (*r =* 0.04, *p* < 0.01) and living with one parent (*r =* 0.07, *p <* 0.001) were marginally more prevalent among newcomers. The 27% attrition at T2 consists of 102 newcomers and 1588 established students (i.e., 6.42% of the participants lost to follow up were newcomers). Missingness ranged from 0 to 19.40% at T1 and up to 27.31% at T2. Additional analysis showed that being lost to follow-up at T2 was slightly more prevalent among those who reported being newcomers (*β =* 0.04 *p <* 0.01), having an immigrant background (*β* = 0.11, *p* < 0.001), or living with one parent (*β* = 0.04, *p* < 0.01), and less prevalent among students who reported having good friends in the classroom (*β*=-0.03, *p* < 0.05). Other predictors (or peer victimization at T1) did not correlate with missingness at T2.

**Table 1:** Descriptive Statistics of study variables (Study 1)

Variable	Missing %	*n*		(%) Mean	SD.	Min	Max	
Newcomer	9.63	5602		(4.77)	0.21	-	-	
Name calling T1	9.58	5605		0.49	0.9	0	4	
Physical T1	9.60	5604		0.23	0.64	0	4	
Excluded T1	9.60	5604		0.34	0.77	0	4	
Rumors T1	9.60	5604		0.28	0.7	0	4	
Name calling T2	27.28	4508		0.48	0.94	0	4	
Physical T2	27.31	4506		0.21	0.71	0	4	
Excluded T2	27.31	4506		0.34	0.83	0	4	
Rumors T2	27.31	4506		0.29	0.76	0	4	
Boy	-	6199		(49.36)	0.5	-	-	
Externalizing behavior	5.45	5861		0.06	0.11	0	0.91	
Lives with one parent	9.57	5606		(24.21)	0.43	-	-	
Immigrant background	19.41	4996		(8.25)	0.28	-	-	
Social anxiety	12.08	5450		1.08	0.85	0	4	
Good friends in the classroom	11.84	5465		(84.54)	0.36	-	-	
Age T1	0.19	6187		12.53	2.09	8.63	19.39	

*Note N* = 6199

According to Little’s missing completely at random (MCAR) test, the missingness was not MCAR but in order to further support the missing at random (MAR) assumption, we included a wide set of auxiliary variables in the analysis and used full information maximum likelihood estimation (FIML) to handle the missingness. To improve the accuracy of the estimates and strengthen the assumptions underlying the MAR assumption, we included 118 auxiliary variables (Enders, 2008). We used an inclusive auxiliary variable strategy, which suggests including numerous variables that either correlate with (Y) and/or missingness (Collins et al., 2001). The included items were measured in all three waves and considered students’ perception of their teacher (1 item x 3), classroom climate (3 items x 3), academic self-concept (2 items x 3) general liking of school (3 items x 3), general perception of peers (12 items x 3), school climate (5 items x 3) as well as their self-reported depression symptom (5 items x 3), self-esteem (5 items x 3), learning difficulties (3 items x 3) and a variable assessing their previous classroom mobility before the data collection (1 item x 1).

### Measures

Children reported their gender (dummy-coded as girl = 0; boy = 1), age in years and months, and whether they lived with both parents (dummy-coded as not living with one parent = 0; living with one parent = 1).

*Immigrant background* refers to a person who was born outside of Finland or whose (one or both) parents were born outside of Finland. In this data, children had their immigrant background from Sweden (23%), Russia (16%), Estonia (8%), United States (2%), Somalia (2%), Far East (6%), Middle East (4%), other European countries (5%), and “elsewhere” (35%). The variable was dummy-coded (not immigrant background = 0; immigrant background = 1).

*Newcomer status* was generated from the questions “Are you a new student in this classroom?”, “In which grade did you enter this classroom?” and “What is your current grade level?” at T1. If the student was a new student in the classroom, and had entered the classroom in their current grade, they were identified as newcomers (scoring 1); others were considered established students (scoring 0). As the questionnaire was filled in at the end of the school year (May), the newcomers had been in their current (i.e., new) classroom for 0—9 months at T1.

*Peer victimization* (T1 and T2). After being provided a definition of bullying as intentional and repeated hurtful behavior towards someone who finds it difficult to defend oneself, the students were asked: How often have you been bullied like this under the last two months? “I was called nasty names or laughed in my face or hurt by insults”; “I was left with no attention or outside all things or all company by my classmates”;” I was hit, kicked, or pushed”; “Other pupils tried to get others hating me by gossiping and telling lies about me” The answers were given on scale from 0 to 4: (0 = not at all; 1 = once or twice; 2 = 2–3 times a month; 3 = once a week; 4 = several times a week (T1 *α* = 0.76; T2 *α* = 0.82)).

The four victimization items were used as indicators of the *latent dependent variable (peer victimization)* at T1 and T2. To measure whether the factorial decisions were valid for the analyses, we ran a confirmatory factor analysis (CFA), to confirm the fit of our measurement model of victimization. The fit of the one-factor model was good at T1 (*χ*^*2*^(2) = 3.41; *RMSEA* = 0.01; *CFI* = 1.00; *TLI* = 1.00; *SRMR* = 0.01) and at T2 (*χ*^*2*^(2) = 19.14; *RMSEA* = 0.04; *CFI* = 0.99; *TLI* = 0.97; *SRMR* = 0.02). We proceeded by testing for factorial invariance across time. The invariance reached the strict invariance, as the change in *CFI* is not equal or more than −0.010, neither is it supplemented by a change ≥ 0.015 in *RMSEA* or a change of *≥.*030 in *SRMR* (see recommendations for large sample sizes by Chen, 2007, pp 501). Thus, the final SEM model was built on a strict invariance model, where factor loadings, intercepts, and residuals were set equal at both time points.

*Externalizing behavior* was assessed by bullying behavior based on peer reports, drawn from the Participant Role Questionnaire (Salmivalli et al., 1996). Participants were asked to nominate classmates who matched the provided descriptions, with no limit on the number of nominations for each of the following behaviors: (1) starts bullying; (2) makes the others join in the bullying; (3) always finds new ways of harassing the victim. Thereafter, proportion scores were calculated dividing the number of received nominations by the number of participants on the class. An average score across the three items was computed, demonstrating high internal consistency (α = 0.91). Proportion scores ranged from 0 to 0.91 (*M* = 0.06, *SD* = 0.11).

*Social anxiety* was assessed with four items based on the *Social avoidance and distress scale* (La Greca & Lopez, 1998; García-López et al., 2001): “I stay quiet when I´m in a group of people”; “I´m afraid of asking others to do things with me as they might turn me down and don’t do things with me”; “I feel quite shy even among those mates I know well”; “It´s difficult for me to ask others to do things with me”, on a scale from 0 to 4 (0 = not at all, 4 = all the time). Social anxiety score was calculated as an average across the items (*α* = 0.79).

*Having good friends in the classroom* was assessed by asking students to report the extent to which they agreed or disagreed with the item: “I have good friends in my classroom” (options ranging from 0 = strongly disagree to 4 = strongly agree). Response options 3 and 4 (indicating agreement or strong agreement) were coded as 1 (has good friends in the classroom) whereas other responses were coded as 0 (no good friends in the classroom).

### Analytic Plan

Due to the high missingness at T2, we investigated whether non-participation at T2 correlated with any of the study predictors. Thereafter, multivariate analyses were performed using Mplus 8.6. version (Muthén & Muthén, 1998–2017) with structural equation modelling (SEM). SEM enables combining path analytic and unobserved latent variable techniques. For model fit, following measures were relied upon: Comparative Fit Index, *CFI* (> 0.95), Tucker-Lewis Index, *TLI* (> 0.95), Root Mean Square Error of Approximation, *RMSEA* (< 0.07), and Standardized Root Mean Square Residual, SRMR (< 0.08).

To properly address potential concerns related to the nested data structure, additional tests were run to investigate whether the association of interest depends on conducting a single-level or multilevel analysis and whether there is heterogeneity in the association of interest at the classroom and/or school level (i.e., random slope effect). A three-level model including classroom and school levels was conducted, testing whether the slope of victimization on newcomer status varied at these levels. Results showed that the association was not dependent on the analytic approach, and the strength of the slope of mobility predicting newcomer victimization did not significantly vary at the classroom or school levels. Intraclass correlations (ICC) for the main predictor and outcome variables ranged from 0.02 to 0.04 at the classroom level and from 0.01 to 0.03 at the school level, indicating small between-group variance. However, we decided to control for the nested data structure using cluster-robust standard errors.

Missingness was handled with FIML together with a set of auxiliary variables. This combination reduces parameter bias (Enders, 2008) and is more efficient than listwise deletion, pairwise deletion, and similar response pattern imputation when handling missing data in SEM (Enders & Bandalos, 2001).

## Results

### Association Between Newcomer Status and Victimization

First, we tested whether being a newcomer was associated with a higher level of victimization, when controlling for the nested nature of the data, gender, age, living with one parent, immigrant background, externalizing behavior, social anxiety, and having good friends in the classroom (Model 1 in Table 2). The model fit was good (*χ*^*2*^(73) = 358.99, *p <* 0.001; *RMSEA* = 0.03; *CFI* = 0.97; *TLI* = 0.95 *SRMR* = 0.03). Newcomer status was associated with victimization at T1 (*B* = 0.37, *p* < 0.001, $\beta$*<*0.2 indicating a small effect, see Acock, 2014) but no more at T2. However, as newcomer status was associated with victimization at T1, we tested its indirect association (via T1 victimization) on T2 victimization using bias-corrected bootstrapping with 1000 draws. The indirect association was also small (*B* = 0.17) but statistically significant (CI 95%= 0.08–0.29), indicating that some of the victimized newcomers continued being victimized one school year later.

**Table 2:** Newcomer status predicting peer-victimization among basic education students, results of two structural equation models (Study 1)

	Model 1							Model 2			
	T1					T2		T1		T2	
	*β (S.E.)*		*B (S.E.)*			*β (S.E.)*	*B (S.E.)*	*β (S.E.)*	*B (S.E.)*	*β (S.E.)*	*B (S.E.)*
Newcomer	0.07** (0.02)		0.37*** (0.10)			0.01 (0.02)	-0.03 (0.12)	0.24** (0.07)	1.22** (0.36)	0.06 (0.09)	0.36 (0.53)
Victimization T1	-		-			0.41***(0.03)	0.48***(0.04)	-	-	0.41***(0.04)	0.47***(0.04)
Gender	0.03 (0.02)		-0.03 (0.04)			-0.004 (0.02)	-0.01 (0.04)	-0.01 (0.02)	-0.02 (0.04)	-0.003 (0.02)	-0.01 (0.04)
Externalizing behavior			1.09***(0.18)			0.12***(0.03)	1.36***(0.30)	0.11***(0.02)	1.09***(0.18)	0.12***(0.03)	1.37***(0.31)
Age	-0.13***(0.02)		-0.06***(0.01)			-0.08***(0.02)	-0.05***(0.01)	-0.11***(0.02)	-0.06***(0.01)	-0.08***(0.02)	-0.05***(0.01)
Lives with one parent	0.02 (0.02)		0.04 (0.04)			0.000 (0.02)	0.000 (0.05)	0.02 (0.02)	0.04 (0.04)	0.01 (0.02)	0.02 (0.05)
Immigrant background	0.04* (0.02)		0.11 (0.08)			0.07** (0.02)	0.32** (0.11)	0.03 (0.02)	0.10 (0.08)	0.08** (0.02)	0.36**(0.11)
Social anxiety	0.26***(0.02)		0.35***(0.02)			0.02 (0.02)	0.02 (0.03)	0.26***(0.02)	0.34***(0.02)	0.02 (0.02)	0.02 (0.03)
Good friends in class	-0.23***(0.02)		-0.67***(0.06)			-0.01 (0.02)	-0.03 (0.08)	-0.20***(0.02)	-0.61***(0.06)	-0.01 (0.02)	-0.02 (0.08)
Newcomer × Good friends in class	-			-		-	-	-0.21** (0.06)	-1.16** (0.34)	-0.004 (0.07)	-0.03 (0.45)
Newcomer × Age	-			-		-	-	0.03 (0.02)	0.06 (0.05)	0.02 (0.03)	-0.39 (0.31)
Newcomer × Social anxiety	-			-		-	-	0.04 (0.02)	0.21 (0.13)	0.01 (0.02)	0.05 (0.07)
Newcomer × Immigrant background	-			-		-	-	0.02 (0.02)	0.21 (0.29)	-0.04 (0.03)	0.05 (0.15)
Newcomer × Lives with one parent	-			-		-	-	0.01 (0.03)	0.07 (0.21)	-0.05 (0.04)	-0.59 (0.46)
Newcomer (indirect)	VIA					0.03** (0.01)	0.17** (0.05)				

*Note N* = 6,065. Model 1: T1 R^2^ = 0.17; T2 R^2^ = 0.20. Model 2: T1 R^2^ = 0.18; T2 R^2^ = 0.20. β = Standardized coefficient, B = Unstandardized coefficient. *p < 0.05, **p < 0.01, ***p < 0.001

**Fig. 2:**
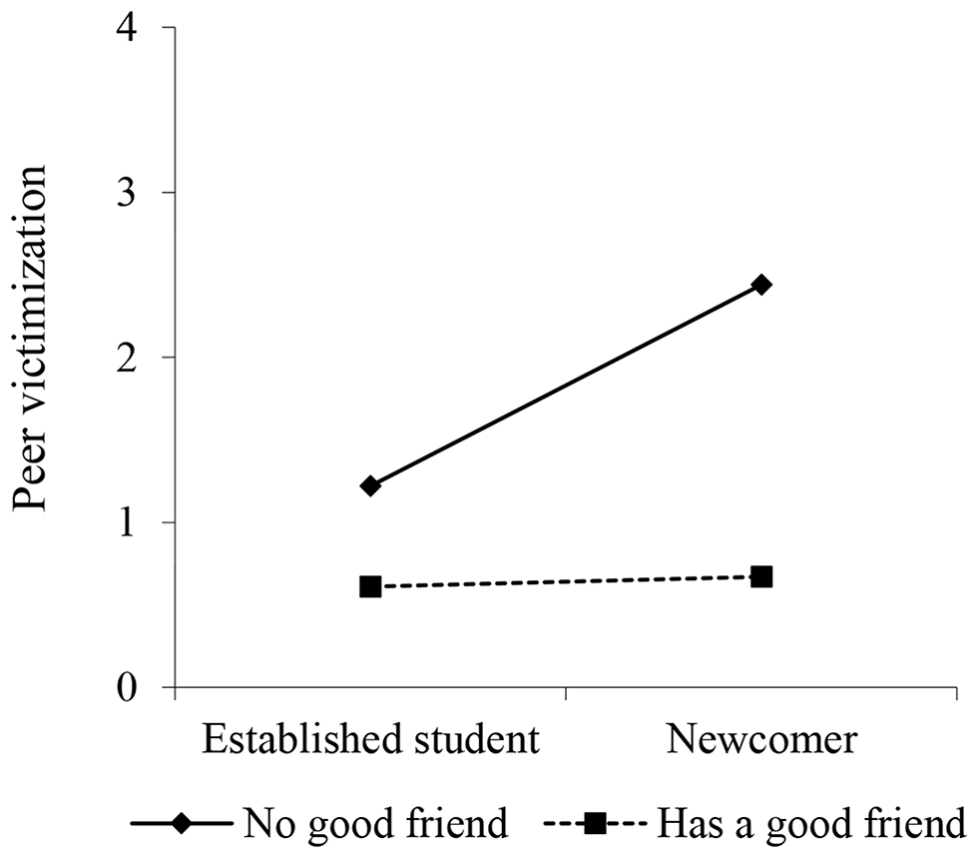
Illustration of the statistically significant interaction (Study 1)

Subsequently, we tested potential moderators (Model 2 in Table 2). The model fit was good (*χ*^*2*^(103) = 401.12, *p <* 0.001; *RMSEA* = 0.02; *CFI* = 0.97; *TLI* = 0.94; *SRMR* = 0.02) and having good friends in the class was the only statistically significant moderator with small effect size $\beta$*>*0.2 (Acock, 2014). Newcomer status increased the risk only among those who did not have good friends in the classroom (slope for “No good friends” *B =* 1.22, *p <* 0.001; “Has a good friend” *B =* 0.06, not sig.) *(*see Fig. 2). In total, 79% of newcomers and 85% of established students reported having friends. None of the other factors tested (age, living with one parent, immigrant background, or social anxiety) significantly moderated the association between newcomer and victimization. Cohen’s *f*^2^ > 0.15 indicated medium collective effect sizes for both models in Table 2 (Cohen, 1992). We further tested each moderator individually within the model, yet the results remained unchanged. Additionally, a sensitivity analysis was conducted on the entire sample while controlling for the intervention, but the results remained unaltered.

## Discussion

We investigated the association between being a newcomer and peer victimization at two time points: in the school year when students entered the new classroom, and the following year. We controlled for gender, externalizing behavior, age, living with one parent, immigrant background, social anxiety, and having good friends in the classroom and tested if the latter five moderated the association between being a newcomer and victimization. As hypothesized (H1), newcomers reported more victimization compared to established students in the year when they had entered the new classroom, and indirectly via this enhanced risk, they were still somewhat more likely to be victimized in the next school year. Only one of the tested moderators affected the association between being a newcomer and victimization: having good friends in the classroom protected newcomers against peer victimization. This suggests that being able to make friends in the new classroom (or maybe have a good friend there already when entering the group) may play a protective role against victimization.

The absence of a direct, long-term association between newcomer status and victimization (in line with H2), along with the significant indirect association, suggests that ingroup favoritism (Tajfel & Turner, 1986) and being “socially misfit” (Wright et al., 1986) in the eyes of established students affects those who have recently entered the classroom. Once victimized, these students remain at a heightened risk even a year later. However, finding a good friend may accelerate integration. Friends can protect newcomers from victimization by helping them get acquainted with group norms, assisting them to become “compatible” group members through peer influence (Laursen & Veenstra, 2021) as well as showing the others they fit in.

Something to consider while interpreting the results is data attrition, as it was found that newcomers were slightly more likely to be lost to follow-up at T2. The newcomers who were lost to follow-up the following year (T2) had higher peer victimization scores (average of four peer-victimization items at T1) (*M*_vic1_=0.62) compared to both newcomers who participated (*M*_vic1_=0.59) and established students who participated (*M*_vic1_=0.33) as well as established students who were lost to follow-up (*M*_vic1_=0.35). This indicates that student mobility seems to be more prevalent among victimized students, being in line with Carson et al. (2013) but also underlining the importance of effective classroom integration of newcomers to avoid further classroom transitions related to peer relation challenges.

Surprisingly, age, living with one parent, immigrant background, or social anxiety did not moderate the association as we expected – even if some of these factors had main effects on victimization. Further studies are needed to understand for which students mobility matters the most. One potentially relevant factor is *why* student mobility occurred. As this could not be tested in Study 1, we conducted another study to shed further light on whether newcomers’ victimization is affected by the reasons for mobility.

## Study 2. Do Reasons of Mobility Explain the Association Between Newcomer Status and Victimization?

Although Study 1 considered some characteristics often associated with student mobility, in Study 2, we delve deeper into the initial reasons of student mobility as well as their role in newcomers’ peer victimization. First, we investigate whether the association between newcomer status and peer victimization replicates in a new sample, and whether this association is moderated by student’s grade level. Subsequently, we assess the reasons for being a newcomer to disentangle which ones of them, if any, drive the hypothesized association. In other words, we examine whether student mobility in general leads to more peer victimization, or whether the outcome differs based on the reason for the mobility.

## Methods

### Participants and Procedures

The data come from a student survey completed annually in schools implementing the KiVa antibullying program in Finland. In the 2022 survey, questions on student mobility were included in the surveys for grades 4–9 (ages 10–15; for younger children, the survey is kept short and simple, and therefore their burden was not increased with these new questions).

In total, 58,936 students from grades 4 to 9 participated in the survey, yet some were excluded from the study sample. In Finland, active parental consent is not required for large-scale surveys that are cunducted as part of a school’s routine activities. First, 44 participants were excluded due to contradictory answers to the initial reasons for student mobility (moving with the family and moving because of parental dissolution), and then 154 cases were excluded due to missing data on all variables. The final analytical sample consisted of 58,738 students. Among them, 4.5% were newcomers, and moving with the family was the most common reason for changing schools (Table 3).

**Table 3:** Descriptive statistics of study variables (Study 2)

Variable	Missing %	*n*	% (Mean)	SD	Min	Max	
Newcomer	-	58,738	4.51	0.21	-	-	
* Moved with family*	-	58,738	2.20	0.15	-	-	
* Parental dissolution*	-	58,738	0.22	0.05	-	-	
* Previous victim*	-	58,738	0.35	0.06	-	-	
* Previous bully*	-	58,738	0.04	0.02	-	-	
* More suitable school*	-	58,738	0.72	0.08	-	-	
* Other*	-	58,738	1.48	0.12	-	-	
Name calling	0.14	58,656	(0.31)	0.80	0	4	
Physical	0.43	58,484	(0.17)	0.60	0	4	
Excluded	0.25	58,589	(0.26)	0.74	0	4	
Rumors	0.35	58,533	(0.21)	0.66	0	4	
Grade level	-	58,738	(6.26)	1.68	4	9	

*Note N* = 58,738

### Measures

*Grade level* (4 to 9) was reported by the participants.

*Newcomer status* was assessed with the following question: “Are you a new student in this school? (Have you transferred to this school from another school during the current school year, i.e., after last summer break?)”, with options: (1) No. I’m in the same school as in the previous year; (2) Yes. Only I changed schools, not the other classmates; (3) Yes, because most (or all) of the students my age transferred to this school last autumn; (4) Yes, most (or all) of the students transferred to a new school, but I transferred to a different school than others. For the purposes of the current study, participants who chose the option 2 or 4 were considered *newcomers*. As the data collection was conducted in spring semester (March), the newcomers had been in their current classroom for 0—7 months.

The following questions (only asked from newcomers) addressed the *initial reason of mobility*: Why did you change to this school? (You can tick all the options that apply): My family relocated, and thus my school changed; My parents separated, and I relocated to a new home with one parent; I was bullied in the previous school/classroom; I bullied others in the previous school/classroom; This was a more suitable school for me; Something else; I don’t know. The last two options were combined into “other”. Participants were only coded as responding “other” if they did not report another, more informative reason for mobility. Due to multiple choice categories, some overlap is present. For example, 63 participants (5.12%) of those who reported “moved with a family” also reported being “victimized in the previous school,” and five (0.39%) reported they “bullied others in the previous school.” Robustness check was later conducted using a restricted sample (excluding those with overlapped responses.)

*Peer victimization* was assessed with four items representing name-calling, social exclusion, physical victimization, and rumor-spreading ($\alpha$=0.79). Students responded, on a scale from 0 to 4 (0 =not at all; 1 = once or twice; 2 = 2–3 times a month; 3 = once a week; 4 = several times a week) to the question: How often have you been bullied like this during the last two months? I was called names, mocked, or teased in a hurtful way; The other students ignored me completely or excluded me from the group; I was hit, kicked, or pushed; The other students spread mean or offending stories about me.

### Analytic Plan

First, we present the descriptives of the study variables, including missingness. CFA with four observed victimization variables indicated an excellent fit for a one-factor model (*χ*^*2*^(2) = 32.81, *p <* 0.001; *RMSEA* = 0.02; *CFI* = 1.00; *TLI* = 1.00; *SRMR* = 0.01). Thereafter, the multivariate analyses are conducted using SEM. As in Study 1, the nested nature of data (students within schools) is adjusted for. Overall, the data set did not include many missing values, but we used FIML estimation during the analysis (missingness on study variables < 1%). Mplus 8.6 version (Muthén & Muthén, 1998–2017) is applied to estimate SEM models. Fit indices (i.e., *CFI* > 0.95, *TLI* > 0.95, *RMSEA* < 0.07 and *SRMR* < 0.08) were utilized for evaluating the model fit.

## Results

The results of the multivariate analysis are reported in Table 4. The first model (Model 1) had an excellent fit (*χ*^*2*^(8) = 184.59, *p <* 0.001; *RMSEA* = 0.02; *CFI* = 0.99; *TLI* = 0.99; *SRMR* = 0.01), and it showed that newcomer status was positively associated with peer victimization (*B* = 0.15, *p* < 0.001, $\beta$<0.2 i.e., small effect, see Acock, 2014). Thus, newcomers tended to report slightly higher peer victimization compared to established children. Age did not moderate this association.

**Table 4:** Newcomer status and reasons for mobility predicting peer-victimization. Two models presented in the same table with standardized and unstandardized coefficients (Study 2)

Peer-victimization	Model 1			Model 2	
	*β (S.E.)*		*B (S.E.)*	*β (S.E.)*	*B (S.E.)*
Newcomer	0.06***(0.01)	0.15***(0.02)		-	-
*Moved with family*				0.02** (0.01)	0.06** (0.02)
*Parental dissolution*				0.02** (0.01)	0.17** (0.06)
*Previous victim*				0.08***(0.01)	0.67***(0.09)
*Previous bully*				0.05***(0.01)	1.31***(0.26)
*More suitable school*				0.00 (0.01)	− 0.00 (0.04)
*Other reason*				0.04***(0.01)	0.15***(0.03)

*Note N* = 58,738. Both models are adjusted for grade level and clustered by school ID. β = Standardized coefficient, B = Unstandardized coefficient. *p* < 0.05*, *p* < 0.01**, *p* < 0.001

The second model (Model 2) had an excellent fit as well (*χ*^*2*^(23) = 226.61; *RMSEA =* 0.01; *CFI* = 0.99; *TLI* = 0.99; *SRMR* = 0.01), and indicated that all the initial causes of mobility, except “changing to a more suitable school” were associated with higher peer victimization compared to students who did not change schools. These effect sizes were small (Acock, 2014). Comparing standardized estimates of the mobility causes on peer victimization, Model 2 shows that of all reasons, changing schools due to former peer victimization was the strongest predictor of victimization in the new school, followed by changing schools because of bullying others, followed by “other reason”. Both types of residential mobility also predicted slightly higher victimization score compared to established students.

## Discussion

Study 2 examined the association between newcomer status and peer victimization among a recently collected sample of Finnish basic education students and delved into the initial causes of student mobility to investigate whether any particular reason for mobility drives the association over others. The result of newcomer status predicting victimization was replicated in this new sample and again, it was not moderated by age (now operationalized as grade level). Furthermore, it was found that all reasons for mobility – except for changing to a more suitable school – predicted higher victimization compared to established students. Changing to a more suitable school (e.g., weighted, or special teaching) showed no link to victimization, likely because students in these classrooms may have entered them simultaneously, as well as be more similar in their family backgrounds, interests, and abilities. The 95% confidence interval levels showed a statistically significant difference between those who had changed schools due to residential moves and those with a victimization history. Hence, changing schools due to former victimization seems to have the strongest association with newcomer victimization. Thus, it is important to consider the possibility that some newcomers have pre-existing challenges that contribute to their victimization, rather than solely attributing it to the student mobility. Unfortunately, based on these findings, it is not possible to say if changing schools helps a former victim or not. Although formerly victimized students are victimized in the new context *more than other students*, their victimization trajectory, i.e. whether they are victimized more or less compared with *their own previous level* remains unknown.

### General Discussion

Changing classrooms or schools is a challenging event in children’s lives as they might lose their old friends in the transition, need to adapt to new classroom norms and routines, as well as build their new social relationships from scratch. In addition, student mobility might be a consequence of stressful events occurring in the family or in the previous school (e.g., Carson et al., 2013; Tønnessen et al., 2016). Thus, the current study investigated whether newcomers entering established classrooms were at a heightened risk for peer victimization while considering several family factors and individual characteristics as potential moderators. We further examined if the reason for mobility drives the association rather than the newcomer status as such. The study contributes to student mobility literature by approaching the topic from the peer relations perspective.

The findings indicated that being a newcomer is associated with heightened victimization during the school year of entering the new classroom, and indirectly, even one year later. Although the found effect sizes varied from small to medium, it is crucial not to overlook the potential risk of student mobility, as it can still contribute to a student’s vulnerability to victimization to some degree. The association between newcomer status and victimization during the school year when mobility happened was replicated in two independent samples. In Study 1, this association was moderated by having good friends in the classroom. This is in line with previous research (Hodges et al., 1999) suggesting that having friends can alleviate the risk of victimization. However, many newcomers reported lacking good friends at the end of the school year in which they entered the classroom. As prior research suggests, newcomers tend to have fewer friends, and their roles are less central and prestigious in adolescents’ social networks (South & Haynie, 2004). Thus, it seems like newcomers tend to face difficulties finding their position in already established peer groups, which then amplifies their risk for peer victimization.

The interpretation of the observed cross-sectional moderation and the absence of long-term moderation deserves further exploration. Initially, newcomers experience higher levels of victimization due to their status as incompatible “outsiders” in the eyes of their new peers. That is, being outgrouped by established students may contribute to increased victimization experiences among newcomers. The moderation observed at T1, where having a friend in the classroom was associated with lower levels of victimization for newcomers, supports the idea that social connections can offer protection against victimization, especially for those who are initially perceived as outsiders. Finding a friend may help in the process of becoming compatible with the new peer group. By T2, newcomers have had more time to integrate into the classroom environment, establish social connections, and be exposed to the peer influence further making them more compatible with their peers. As a result, the direct association between newcomer status and victimization may diminish, and the moderation of friendship may no longer be as pronounced because newcomers are no longer perceived as distinct outsiders. Despite the passing of time, the indirect association observed between T1 and T2 victimization suggests that small proportion of newcomers may continue to face difficulties in integrating or may remain vulnerable to victimization. This could be due to gradual changes (Olweus, 1978), where victimization alters how peers perceive the victim. Over time, bullying may become socially normalized and bullies may justify their acts through moral disengagement (Bandura et al., 1996). This maintains the cycle of victimization as peers view the victim as increasingly worthless and deviant from others and justify their harmful behavior with various excuses. Such peer behavior excludes the victim, leading to a persistent outgrouping, friendlessness and victimization.

We proposed that initially newcomer’s difficulties with peers may link to their incompatibility with new classmates. Based on social identity theory (Tajfel & Turner, 1986) we suggested that there is an ingroup bias that contributes to the negative treatment of newcomers who may seem as “incompatible” with established students. As expected, newcomers reported higher victimization during the school year in which they entered the classroom, but there was no direct long-term association one year later (after integration). This finding emphasizes the roles of teachers and established students in the newcomer’s integration process; perhaps more concrete tools are needed to hinder ingroup favoritism in classrooms receiving newcomers.

Furthermore, newcomers’ social environment tends to change substantially during the transition as previous social ties are left behind. Thus, we suggest that newcomer status might influence *the experience of victimization* (e.g., feeling lonely and excluded) with the presence of friendships moderating this relationship. Having friends after the transition indeed alleviated newcomers’ victimization, supporting previous literature on friendships and peer victimization (Hodges et al., 1999). Being able to find friends after the transition hastens the integration (i.e., becoming part of the ingroup), but finding a friend may not be easy as friendships in the new classroom are already established. However, even seemingly casual friendships or a student’s perception of having a friend to spend time with in the classroom can serve as a protective factor. The literature on adolescents’ social networks has shown that newcomers tend to have fewer friends, a tendency to report non-reciprocal best friends as well as be involved in networks whose members are unpopular (South & Haynie, 2004). Therefore, it is critical to be aware of the newcomers’ potential challenges to fit into friendship networks and find ways to achieve common grounds between established students and newcomers.

Lastly, we proposed that additional vulnerabilities related to student mobility would exacerbate the newcomers’ victimization. The results did not support this assumption. Living with a single parent, immigrant background or social anxiety did not moderate the association. Moreover, all reasons of mobility measured, except changing to a more suitable school, predicted higher victimization among newcomers as compared with established peers. Yet, one fragility stood out in Study 2: the newcomers who had been previously victimized reported significantly higher victimization in new settings compared to other newcomers. This finding signals that victimization is also associated with somewhat stable characteristics that follow a student along the transition (see also Salmivalli et al., 1998). Previously victimized children in particular need help in integrating into peer groups and finding their position in friendship networks. We encourage further studies to test whether these vulnerabilities cumulatively impact newcomer victimization. This approach was not feasible in this study due to small cell sizes.

Reflecting on the supported mechanisms above, one may argue that finding a friend possibly moves the newcomer into the established peer group (ingroup), while those newcomers without friends or those who bond with less popular students (perhaps also perceived as outgroup members) remain in the outgroup, increasing their risk for victimization. Further studies on newcomers would benefit from the investigation on how intergroup contact and bias predict newcomer’s integration and peer victimization. In addition, we assume that the newcomer’s integration into the class and finding friends may be related to other factors as well. For example, a child’s prior individual experiences may influence their integration into the new classroom and getting along with new peers. Family adversity, for instance, may lead to difficulties with peers and a lack of parental support in cases of peer victimization. Also, a family’s socioeconomic status may alleviate or exacerbate the integration into a new classroom. Thus, further studies on newcomers would also benefit from focusing on such family-level factors, as peer victimization seems to follow the individual across transitions to some extent.

Disentangling the risk factors is needed so that parents and school professionals can more effectively support children who are most likely to face difficulties as newcomers. In addition, future research should focus on tailored newcomer interventions, especially for students who have already experienced victimization in their previous school and who are, based on our findings, most at risk. However, this is not to say that these students should be targeted by individual interventions. Rather, approaches involving the whole classroom, encouraging interaction and inclusion while minimizing ingroup favoritism might be advisable.

Our finding that student mobility predicts peer victimization stands in contrast to some previous research, which might be explained by contextual and methodological differences. For example, Carson et al. (2013) compared the victimization scores before and after the mobility within the same school district, finding a decrease in peer victimization. The lack of statistically significant association in the study by Vandell et al. (2021) might be attributed to the relatively high proportion of newcomers in their sample, compared to our studies (17% in Carson et al.; 4.77% and 4.45% in the current samples), rendering the event more normative in their sample. In the Finnish context, there is less mobility and students have often stayed with the same classmates for long periods of time. Norford and Medway (2002) explored the initial reasons for mobility (including many family-related reasons but not previous peer victimization) on depression and social involvement, finding only parental separation as a predictor of lower participation in extracurricular activities. Also, due to their specific focus on frequent moves and longitudinal outcomes, the strict exclusion criteria (e.g., excluding recent movers and those who had moved less than three times in their lives) may have contributed to the results.

### Strengths and Limitations

The current studies had several strengths. Both involved large samples (Study 1: *n* = 6,199; Study 2: *n* = 58,738) which is especially important when studying newcomers, who represent a minority. The combination of Study 1 and Study 2 enabled an in-depth examination of our research question and provided an opportunity to replicate the finding regarding newcomers’ victimization. Non-response bias may influence the findings if the respondents and non-respondents differ from each other by their characteristics. Yet, in this study, the questionnaires were filled during teacher-led sessions, minimizing this bias.

However, there were also limitations that should be noted. First, something to consider in Study 1 is the amount of missing data. To understand the characteristics of participants lost to follow-up, we examined which predictors were associated with not completing the survey at T2. Newcomer status, living with one parent, having an immigrant background, and not having a friend in the classroom were all predictors of not completing the survey. Thus, it is possible that some of the data attrition can be attributed to frequent mobility, as mobility is an event that tends to accumulate within certain families. For example, previous literature has highlighted that children living in single-headed households (Pribesh & Downey, 1999) and families with an immigrant background (Kuyvenhoven & Das, 2022; South & Haynie, 2004) tend to be overrepresented among mobile students.

Another limitation is the possibility of unobserved variables influencing the findings. Previous research has emphasized that initial differences, including individual and family level characteristics, between newcomers and established students are essential to consider when examining the differences between these groups (Calibuso & Winsler, 2020). Although we controlled for several of these characteristics in Study 1, the possibility of an unobserved confounder, such as socioeconomic status, cannot be ruled out. In addition, as Study 1 did not separate between classroom-, school-, and resident changes, we need to consider the possibility that the associations may depend on the extent of the change (i.e., whether the child changes classrooms, schools, residents, or all of these at the same time). In Study 2, we had a limited number of variables in the data set and thus could not control for many confounders. Another issue that needs to be considered when interpreting the results of Study 2 is that only newcomers replied to questions about reasons for mobility, while also some of the established students may have experienced similar events (such as parental dissolution or peer victimization in previous school years).

Additionally, the exact time of the transition was unknown; the newcomers had entered the classroom in the year of data collection, but it is uncertain whether that happened at the beginning of the autumn semester or later, potentially influencing the estimates to some extent. However, if anything this is likely to underestimate the association between newcomer status and victimization, as some integration might already have happened among those who had spent up to nine months in the new peer context (as compared with those who had arrived recently).

Despite the large datasets used in this study, some details need to be considered: While the data in Study 1 included only control schools from an evaluation trial of a bullying prevention program, the data in Study 2 was collected in schools implementing the program. Sensitivity analysis was performed in Study 1 using the whole sample and controlling for the intervention (which did not change the key findings). The findings show that newcomers are at a heightened risk for victimization even in schools implementing a whole-school bullying prevention program. More tailored actions might be needed to tackle newcomer victimization.

Also, it is essential to note that our study relies solely on self-reports. Our rationale behind using self-reports is to emphasize the students’ subjective experiences of peer victimization, valuing their perspectives in understanding the phenomenon. Commonly known limitations for self-reports in peer victimization studies are recall bias as well as victims’ hesitance to disclose victimization experiences. Similarly, we acknowledge the subjectivity inherent in asking about students’ friendships; however, we find that a simple question about having friends in the classroom is not only easily understood but also interpretable within our context, which increases this measurement’s face validity.

Finally, the Finnish context is relatively unique, which can affect the generalizability of the findings. Since the topic lacks international comparison, it is difficult to estimate whether these results are extremely or just marginally concerning, as the situation of newcomers may be worse or better in other contexts.

## Conclusions

This study increases our understanding of student mobility among basic education students. As newcomers tended to report higher peer victimization in new classrooms and schools than their established peers, targeted actions to tackle ingroup favoritism and support newcomers’ integration might benefit this potentially vulnerable population. We argue that student mobility should be considered a risk factor for peer victimization among basic education students in the Finnish context. Changing schools because of previous peer victimization had the strongest association with the newcomer’s victimization in a new classroom. This is something school professionals and parents dealing with children’s peer victimization cases should take into consideration: changing classrooms or schools is unlikely to stop victimization if not complemented with other support.
